# Survival associated with extent of radical hysterectomy in early-stage cervical cancer: a subanalysis of the Surveillance in Cervical CANcer (SCCAN) collaborative study

**DOI:** 10.1016/j.ajog.2023.06.030

**Published:** 2023-06-17

**Authors:** Nicolò Bizzarri, Denis Querleu, Lukáš Dostálek, Luc R. C. W. van Lonkhuijzen, Diana Giannarelli, Aldo Lopez, Sahar Salehi, Ali Ayhan, Sarah H. Kim, David Isla Ortiz, Jaroslav Klat, Fabio Landoni, Rene Pareja, Ranjit Manchanda, Jan Kosťun, Pedro T. Ramirez, Mehmet M. Meydanli, Diego Odetto, Rene Laky, Ignacio Zapardiel, Vit Weinberger, Ricardo Dos Reis, Luigi Pedone Anchora, Karina Amaro, Huseyin Akilli, Nadeem R. Abu-Rustum, Rosa A. Salcedo-Hernández, Veronika Javůrková, Constantijne H. Mom, Giovanni Scambia, Henrik Falconer, David Cibula

**Affiliations:** Unità Operativa Complessa Ginecologia Oncologica, Dipartimento per la Salute della Donna e del Bambino e della Salute Pubblica, Fondazione Policlinico Universitario A. Gemelli, IRCCS, Rome, Italy; Unità Operativa Complessa Ginecologia Oncologica, Dipartimento per la Salute della Donna e del Bambino e della Salute Pubblica, Fondazione Policlinico Universitario A. Gemelli, IRCCS, Rome, Italy; First Faculty of Medicine, Department of Obstetrics and Gynecology, Gynecologic Oncology Center, Charles University and General University Hospital (Central and Eastern European Gynecologic Oncology Group), Prague, Czech Republic; Center for Gynaecologic Oncology Amsterdam, Amsterdam University Medical Centers, Amsterdam, the Netherlands; Biostatistics Unit, Scientific Directorate, Fondazione Policlinico Universitario A. Gemelli, IRCCS, Rome, Italy; Department of Gynecological Surgery, National Institute of Neoplastic Diseases, Lima, Peru; Department of Pelvic Cancer, Karolinska University Hospital, Stockholm, Sweden; Department of Women’s and Children’s Health, Karolinska Institutet, Stockholm, Sweden; Division of Gynecologic Oncology, Department of Gynecology and Obstetrics, Baskent University School of Medicine, Ankara, Turkey; Memorial Sloan Kettering Cancer Center, New York, NY; Gynecology Oncology Center, National Institute of Cancerology Mexico, Mexico City, Mexico; Faculty of Medicine, Department of Obstetrics and Gynecology, University Hospital and University of Ostrava, Ostrava, Czech Republic; IRCCS Fondazione San Gerardo - Università Milano Bicocca, Monza, Italy; Department of Gynecologic Oncology, Instituto Nacional de Cancerología, Bogotá, Colombia; Wolfson Institute of Population Health, Barts Cancer Centre, Queen Mary University of London, and Barts Health NHS Trust, London, United Kingdom; Department of Gynaecological Oncology, Barts Health NHS Trust, London, United Kingdom; Faculty of Public Health and Policy, Department of Health Services Research, London School of Hygiene and Tropical Medicine, London, United Kingdom; Department of Gynaecology and Obstetrics, University Hospital Pilsen, Charles University, Prague, Czech Republic; Houston Methodist Hospital, Houston, TX; Department of Gynecologic Oncology, Zekai Tahir Burak Women’s Health and Research Hospital, University of Health Sciences, Ankara, Turkey; Department of Gynecologic Oncology, Hospital Italiano de Buenos Aires, Instituto Universitario Hospital Italiano, Buenos Aires, Argentina; Department of Gynecology, Medical University of Graz, Graz, Austria; Gynecologic Oncology Unit, La Paz University Hospital - IdiPAZ, Madrid, Spain; Faculty of Medicine, University Hospital Brno, Masaryk University, Brno, Czechia; Department of Gynecologic Oncology, Barretos Cancer Hospital, Barretos, Sao Paulo, Brazil; Unità Operativa Complessa Ginecologia Oncologica, Dipartimento per la Salute della Donna e del Bambino e della Salute Pubblica, Fondazione Policlinico Universitario A. Gemelli, IRCCS, Rome, Italy; Oncology Unit, Cayetano Heredia Hospital, Lima, Peru; Division of Gynecologic Oncology, Department of Gynecology and Obstetrics, Baskent University School of Medicine, Ankara, Turkey; Memorial Sloan Kettering Cancer Center, New York, NY; Gynecology Oncology Center, National Institute of Cancerology Mexico, Mexico City, Mexico; Faculty of Medicine, Department of Obstetrics and Gynecology, University Hospital and University of Ostrava, Ostrava, Czech Republic; Center for Gynaecologic Oncology Amsterdam, Amsterdam University Medical Centers, Amsterdam, the Netherlands; Unità Operativa Complessa Ginecologia Oncologica, Dipartimento per la Salute della Donna e del Bambino e della Salute Pubblica, Fondazione Policlinico Universitario A. Gemelli, IRCCS, Rome, Italy; Department of Pelvic Cancer, Karolinska University Hospital, Stockholm, Sweden; Department of Women’s and Children’s Health, Karolinska Institutet, Stockholm, Sweden; First Faculty of Medicine, Department of Obstetrics and Gynecology, Gynecologic Oncology Center, Charles University and General University Hospital (Central and Eastern European Gynecologic Oncology Group), Prague, Czech Republic

**Keywords:** cervical cancer, early stage, laparotomy, radical hysterectomy, radicality, surgery, survival

## Abstract

**BACKGROUND:**

International guidelines recommend tailoring the radicality of hysterectomy according to the known preoperative tumor characteristics in patients with early-stage cervical cancer.

**OBJECTIVE:**

This study aimed to assess whether increased radicality had an effect on 5-year disease-free survival in patients with early-stage cervical cancer undergoing radical hysterectomy. The secondary aims were 5-year overall survival and pattern of recurrence.

**STUDY DESIGN:**

This was an international, multicenter, retrospective study from the Surveillance in Cervical CANcer (SCCAN) collaborative cohort. Patients with the International Federation of Gynecology and Obstetrics 2009 stage IB1 and IIA1 who underwent open type B/C1/C2 radical hysterectomy according to Querleu-Morrow classification between January 2007 and December 2016, who did not undergo neoadjuvant chemotherapy and who had negative lymph nodes and free surgical margins at final histology, were included. Descriptive statistics and survival analyses were performed. Patients were stratified according to pathologic tumor diameter. Propensity score match analysis was performed to balance baseline characteristics in patients undergoing nerve-sparing and non–nerve-sparing radical hysterectomy.

**RESULTS:**

A total of 1257 patients were included. Of note, 883 patients (70.2%) underwent nerve-sparing radical hysterectomy, and 374 patients (29.8%) underwent non–nerve-sparing radical hysterectomy. Baseline differences between the study groups were found for tumor stage and diameter (higher use of non–nerve-sparing radical hysterectomy for tumors >2 cm or with vaginal involvement; *P*<.0001). The use of adjuvant therapy in patients undergoing nerve-sparing and non–nerve-sparing radical hysterectomy was 27.3% vs 28.6%, respectively (*P*=.63). Five-year disease-free survival in patients undergoing nerve-sparing vs non–nerve-sparing radical hysterectomy was 90.1% (95% confidence interval, 87.9–92.2) vs 93.8% (95% confidence interval, 91.1–96.5), respectively (*P*=.047). Non–nerve-sparing radical hysterectomy was independently associated with better disease-free survival at multivariable analysis performed on the entire cohort (hazard ratio, 0.50; 95% confidence interval, 0.31–0.81; *P*=.004). Furthermore, 5-year overall survival in patients undergoing nerve-sparing vs non–nerve-sparing radical hysterectomy was 95.7% (95% confidence interval, 94.1–97.2) vs non–nerve-sparing 96.5% (95% confidence interval, 94.3–98.7), respectively (*P*=.78). In patients with a tumor diameter ≤20 mm, 5-year disease-free survival was 94.7% in nerve-sparing radical hysterectomy vs 96.2% in non–nerve-sparing radical hysterectomy (*P*=.22). In patients with tumors between 21 and 40 mm, 5-year disease-free survival was 90.3% in non–nerve-sparing radical hysterectomy vs 83.1% in nerve-sparing radical hysterectomy (*P*=.016) (no significant difference in the rate of adjuvant treatment in this subgroup, *P*=.47). This was confirmed after propensity match score analysis (balancing the 2 study groups). The pattern of recurrence in the propensity-matched population did not demonstrate any difference (*P*=.70).

**CONCLUSION:**

For tumors ≤20 mm, no survival difference was found with more radical hysterectomy. For tumors between 21 and 40 mm, a more radical hysterectomy was associated with improved 5-year disease-free survival. No difference in the pattern of recurrence according to the extent of radicality was observed. Non–nerve-sparing radical hysterectomy was associated with better 5-year disease-free survival than nerve-sparing radical hysterectomy after propensity score match analysis.

## Introduction

Despite the introduction and implementation of screening and vaccination programs, cervical cancer remains a major burden, being the fourth most frequent cancer diagnosed in women worldwide.^1^ Radical hysterectomy (RH) with sentinel lymph node biopsy and pelvic lymphadenectomy is the standard treatment for patients with early-stage cervical cancer.^2,3^ International guidelines recommend tailoring surgical radicality based on preoperative risk stratification,^2,3^ with the choice of an individual surgeon to further increase radicality, defined according to Querleu-Morrow–modified classification system.^4^ The rationale behind the use of a more radical parametrectomy is driven by the need of removing occult parametrial disease. A more radical surgery is expected to lead to a survival improvement and to reduce the need for adjuvant radiation therapy. However, it is associated with increased intra- and postoperative morbidities.^5,6^

Few studies have investigated the prognostic effect of more vs less RH in early-stage cervical cancer.^5-9^ Of note, 2 randomized trials have failed to show a survival advantage in the more radical surgery group.^5,9^ In contrast, a recent nationwide cohort study demonstrated a survival improvement in patients with larger tumors (>2 cm, particularly if >4 cm) if a more radical (compared with less radical) hysterectomy was performed.^8^

Recently, the Surveillance in Cervical CANcer (SCCAN) consortium has published 2 retrospective studies on the annual recurrence risk model for tailored surveillance strategy in patients with cervical cancer^10^ and on the post-recurrence survival in patients with cervical cancer.^11^

This study aimed to assess whether the extent of RH had an effect on 5-year disease-free survival (DFS) in patients with early-stage cervical cancer, from the cohort previously included in the SCCAN collaborative studies. The secondary aims were to compare 5-year overall survival (OS) and pattern of recurrence.

## Materials and Methods

The SCCAN is an international, multicenter, retrospective cohort study.^10^ The SCCAN study consortium consists of 20 tertiary centers with a large volume of cervical cancer cases from Europe, Asia, North America, or Latin America. The preoperative management of cervical cancer included the use of 1 modern imaging modality in clinical staging (magnetic resonance imaging, expert ultrasound, computed tomography, or positron emission tomography plus computed tomography). Preoperative histology diagnosis of cervical cancer was obtained by punch biopsy or by cervical conization. Cases were discussed by a multidisciplinary team, surgery and histology assessment were performed by gynecologic oncologists and pathologists with experience in gynecologic oncology, and institutional follow-up was performed by physicians.

Patients were included in the SCCAN cohort^10^ if they met the following inclusion criteria: (1) histologically confirmed cervical cancer treated between January 2007 and December 2016; (2) Tumour, Node, Metastasis (TNM) stage T1a to T2b (based on the preoperative assessment; American Joint Committee on Cancer and Cervix Uteri Cancer Staging); (3) primary surgical management, including fertility-sparing procedures or surgical treatment after neoadjuvant chemotherapy; and (4) negative surgical margins. Patients were treated in national referral centers for gynecologic oncology according to updated national and international guidelines. Pathologic tumor diameter was measured as the largest tumor diameter on the hysterectomy specimen or by the addition of the largest tumor diameter on hysterectomy and preoperative conization specimen. For the current substudy, we selected patients with the International Federation of Gynecology and Obstetrics (FIGO) 2009 stage IB1 and IIA1 who underwent type B or C1/C2 RH according to the Querleu-Morrow classification,^4^ did not undergo neoadjuvant chemotherapy, and had negative lymph nodes at final histology. To reduce potential bias, only patients undergoing open RH were included, given the results from a randomized trial, which demonstrated worse survival in patients undergoing minimally invasive RH.^12^

The decision to perform type B or type C1/C2 RH was taken based on the attending surgeon’s preference and adapted to the tumor’s size and preoperative characteristics. In brief, according to the previous description of the RH classification,^4^ type B RH involved the resection of the paracervix at the level of the ureter, whereas type C RH involved the transection of the paracervix at its junction with the internal iliac vascular system (type C1 with and type C2 without the preservation of autonomic nerves).

The protocol was approved by the institutional review board (IRB) of the lead institution (General University Hospital in Prague, Czech Republic) in 2016. IRB approval at the participating sites was a prerequisite for participation. The study was performed following the Declaration of Helsinki.

### Statistical analysis

The Strengthening the Reporting of Observational Studies in Epidemiology guidelines were followed in reporting the results of this study.^13^ Demographics and clinical data were summarized by absolute counts and percentages, and the chi-square test was used to assess associations among categorical variables.

DFS was defined as the time from surgery to relapse or all-cause death, whichever came first. OS was defined as the time interval between the date of surgery and the date of death from any cause. Both intervals were censored at the date of the last follow-up if no event was observed. Recurrence was defined as the return of cancer after initial treatment.

We used the Kaplan-Meier method to estimate the distribution of time to event end points of DFS and OS, and differences among curves were assessed using the log-rank test.^14,15^

Cox regression analysis was performed to estimate hazard ratios (HRs) and their 95% confidence intervals (CIs) and to adjust for baseline risk factors.^16^

Statistical analyses were performed, dividing the entire cohort into nerve-sparing RH (type B and C1) and non–nerve-sparing RH (type C2) groups.

Patients were stratified according to pathologic tumor diameter. A propensity score matching analysis was used to adjust for baseline differences between the group of patients undergoing nerve-sparing and non–nerve-sparing RH; a ratio of 1:1 and the nearest neighbor method were used without replacement and with a caliper of 0.2 standard deviation of the propensity score distribution. Baseline variables used to formulate propensity scores included pathologic tumor diameter, lymphovascular space invasion (LVSI), stage, and age. The IBM SPSS statistical software (version 27.0; BM Corporation, Armonk, NY) and R (version 4.1.2; R Foundation for Statistical Computing, Vienna, Austria; library MatchIt) were used.

## Results

### Patients’ characteristics

**FIGURE 1:**
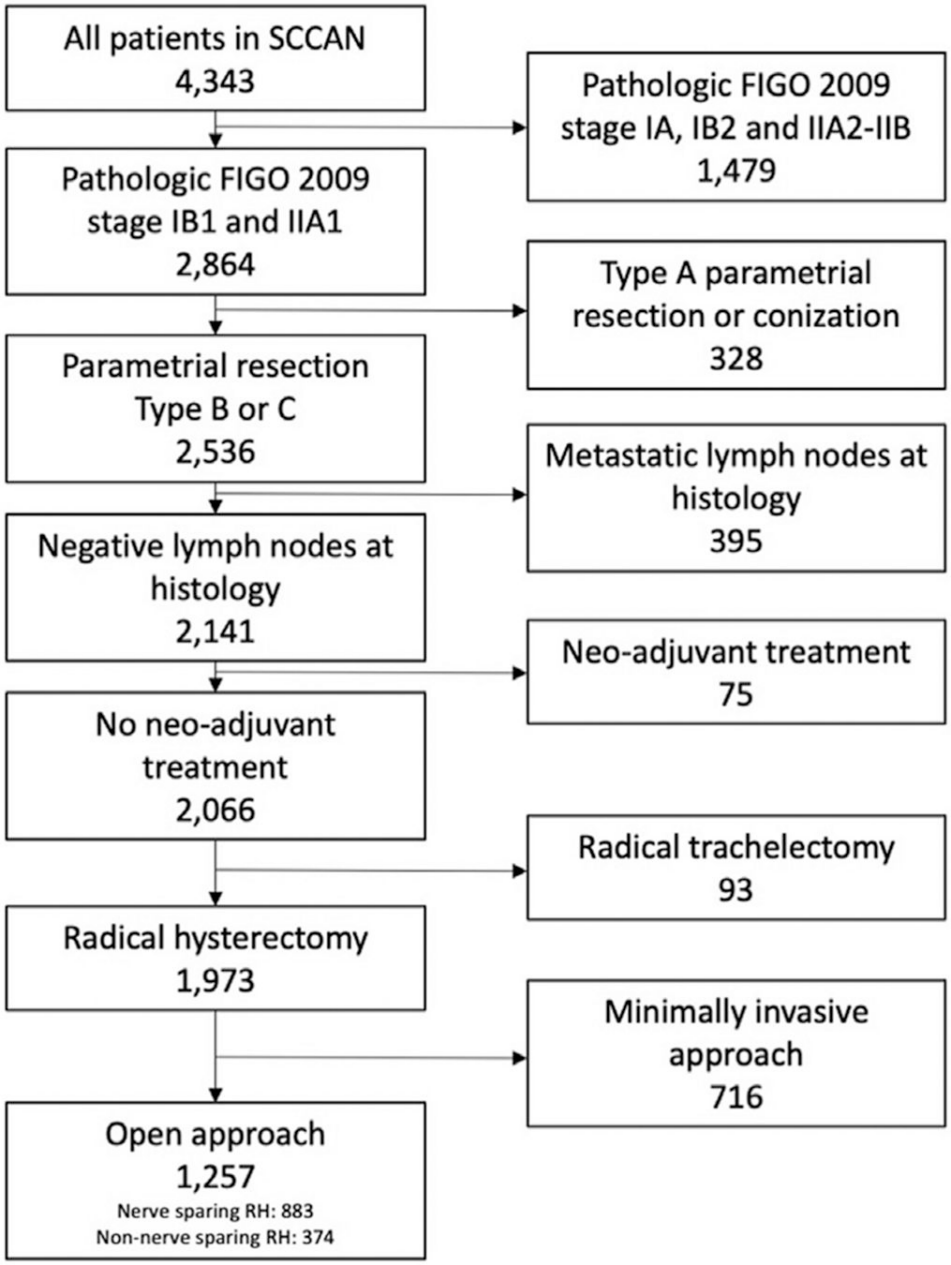
Inclusion and exclusion process *FIGO*, The International Federation of Gynecology and Obstetrics; *RH*, radical hysterectomy; *SCCAN*, Surveillance in Cervical CANcer.

Starting from a database of 4343 patients, we included 1257 patients (28.9%) based on inclusion criteria. The exclusion process is demonstrated in Figure 1. Of the included patients, 883 (70.2%) underwent nerve-sparing RH, and 374 (29.8%) underwent non–nerve-sparing RH. Table 1 shows the clinical and pathologic characteristics of the included patients. Most patients were diagnosed with FIGO stage IB1 (n=1186 [94.4%]), squamous cell carcinoma (n=823 [65.5%]), grade 2 (n=877 [69.8%]), and negative LVSI (n=600 [47.7%]). Most patients did not undergo adjuvant treatment after radical surgery (n=909 [72.3%]).

Baseline difference among the study groups was found in tumor stage and diameter (higher use of non–nerve-sparing RH for tumors >2 cm or with vaginal involvement; *P*<.0001). No difference in the rate of adjuvant therapy was evident between the 2 study groups (*P*=.633).

### Survival analysis of entire population

In the entire cohort (n=1257), the median follow-up time was 5.3 years (interquartile range [IQR], 3.7–7.7). Of note, 5-year DFS in the entire cohort was 91.5% (95% CI, 89.9–93.1), and 5-year OS was 96.0% (95% CI, 94.8–97.2). Moreover, 111 patients (8.8%) had recurrence, and 55 patients (4.4%) died in the entire cohort.

When comparing the 2 groups, a 5-year DFS difference was noted (90.1% [95% CI, 87.9–92.2] in nerve sparing vs 93.8% [95% CI, 91.1–96.5] in nonnerve sparing; *P*=.047). No 5-year OS difference was found (95.7% [95% CI, 94.1–97.2] in nerve sparing vs 96.5% [95% CI, 94.3–98.7] in nonnerve sparing; *P*=.78).

Table 2 demonstrates the Cox multivariable regression analysis for the risk of recurrence in the entire population. Presence of LVSI, histology other than squamous cell, and larger pathologic tumor diameter represented independent risk factors for worse DFS. Supplemental Table 1 shows the Cox multivariable regression analysis for the risk of death in the entire population. No variable independently affected OS (low number of events).

No difference in the pattern of recurrence between the 2 groups was evident (*P*=.99) (Supplemental Table 2).

### Survival analysis according to tumor diameter

**FIGURE 2:**
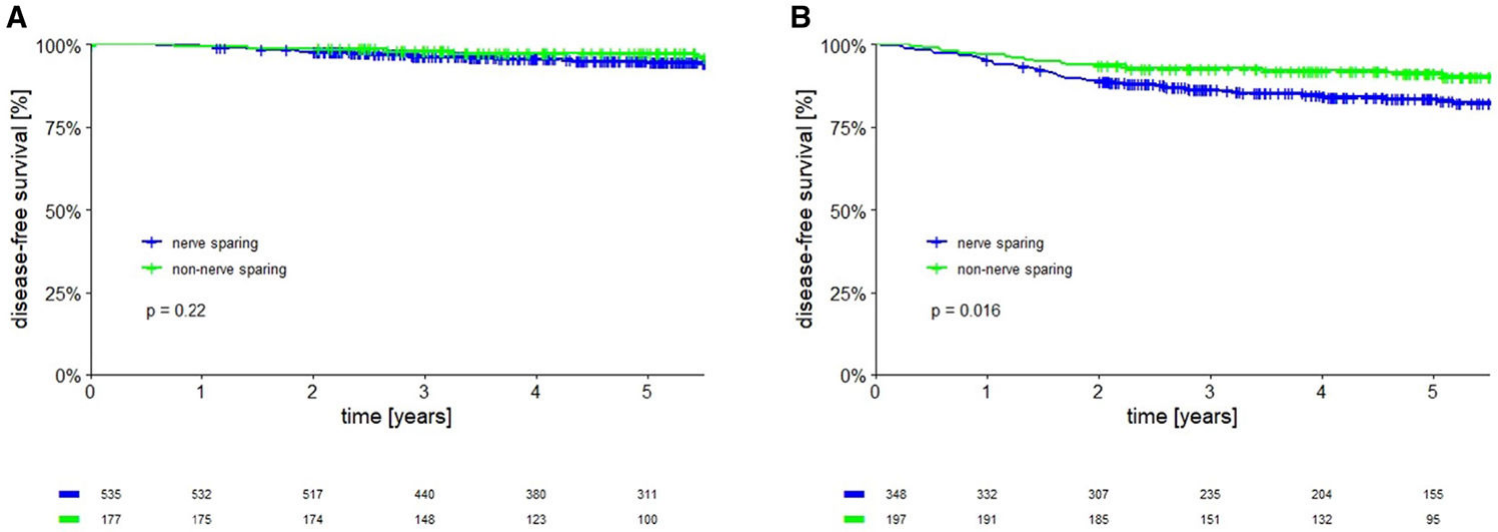
Disease free survival of nerve sparing vs non nerve sparing radical hysterectomy **A,** Patients with tumors ≤20 mm. **B,** patients with tumors between 21 and 40 mm.

Oncological outcomes were evaluated in the 2 groups based on tumor diameter. In patients with tumor diameter ≤20 mm, no 5-year DFS difference was found comparing the 2 groups with different surgical radicality (94.7% in nerve sparing vs 96.2% in nonnerve sparing; *P*=.22) (Figure 2, A). In addition, no difference in the rate of adjuvant treatment administration was noted in this subgroup of patients: 17.8% in nerve sparing vs 17.5% in nonnerve sparing (*P*=.99).

In patients with tumors between 21 and 40 mm, a statistically significant 5-year DFS difference in favor of a non–nerve-sparing approach was noted (83.1% vs 90.3%; *P*=.016) (Figure 2, B). A similar use of adjuvant treatment in patients undergoing nerve-sparing RH (42.0%) vs non–nerve-sparing RH (38.6%) was noted (*P*=.47).

### Propensity match score survival analysis

**FIGURE 3:**
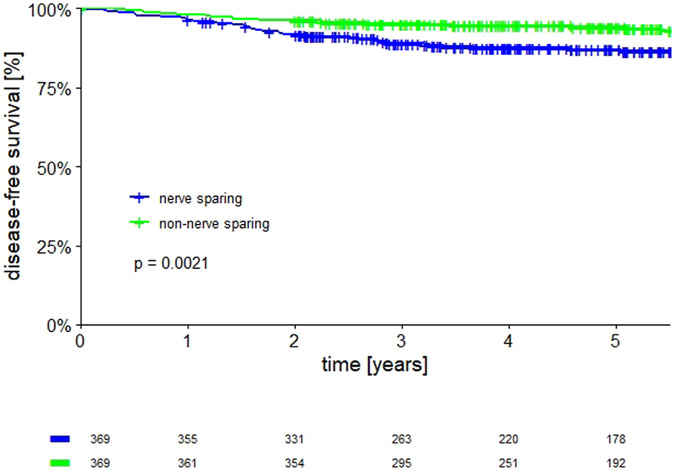
Disease free survival after propensity score matching

To confirm the effect of more extensive RH on survival in the entire cohort of patients, a propensity score matching analysis was performed to balance the baseline characteristics of nerve-sparing and non–nerve-sparing RH groups. After propensity score matching analysis, 369 patients per group were selected with similar clinical-pathological characteristics (Table 3). Most patients had a tumor diameter between 21 and 40 mm (n=387 [52.4%]) and did not receive adjuvant therapy (n=521 [70.6%]). The 5-year DFS was 93.7% (95% CI, 90.9–96.4) in non–nerve-sparing RH vs 86.6% (95% CI, 82.9–90.3) in nerve-sparing RH (*P*=.0021) (Figure 3). The pattern of recurrence in the propensity-matched groups showed no difference between the 2 groups (*P*=.70) (Supplemental Table 3).

## Comment

### Principal findings

In this study, we demonstrated that there was no DFS and OS difference in cervical cancer patients with small tumors (≤20 mm), whereas significantly better DFS was associated with non–nerve-sparing RH in the subgroup of patients with tumor diameters between 21 and 40 mm. Moreover, when nerve-sparing RH was compared with non–nerve-sparing RH in 2 groups with similar baseline characteristics (after propensity score matching analysis), a better DFS was noted in the group treated with a non–nerve-sparing approach.

### Results in the context of what is known

Previous studies analyzed whether the extent of an RH affected the oncologic outcomes in patients with early cervical cancer and reached different conclusions. In particular, 2 prospective randomized trials published by Landoni et al^5^ demonstrated no survival difference when class I was compared with class III (according to the Piver-Rutledge classification) and when class II was compared with class III RH^9^ (of note, the rate of adjuvant treatment in these studies was >50%). Nevertheless, patients who underwent more radical surgery experienced a higher incidence of perioperative complications.^5^ The same results in terms of morbidity were observed by Sun et al^6^ who reported the midterm follow-up results of a randomized trial comparing type II RH vs type III RH in early cervical cancer. These authors concluded that less RH was associated with shorter surgical time, lower intraoperative blood loss, decreased number of postoperative complications, and improved quality of life. In addition, a recent metanalysis showed that nerve-sparing RH (compared with non–nerve-sparing RH) may lessen the risk of postoperative bladder dysfunction, but the certainty of this evidence is low. Moreover, it concluded that the oncological safety of nerve-sparing RH for women with early-stage cervical cancer remains unclear.^17^ The correlation between more RH and impaired quality of life has been shown by other studies not included in the afore-mentioned meta-analysis.^18,19^

In our analysis, non–nerve-sparing RH was associated with improved 5-year DFS at multivariable analysis: this is in contrast with previous data showing that nerve-sparing RH has an equivalent survival outcome to conventional RH.^20^ We could postulate that the survival advantage in non–nerve-sparing RH in our cohort is possibly related to the removal of occult disease in parauterine tissues in larger tumors, which are also at higher risk of perineural invasion,^21^ even though the information on perineural invasion was not documented in our database. Moreover, these results might be explained by the superiority of type C2 RH over C1 and B1, in which the lateral paracervical tissue is not removed.

Further oncological outcomes were reported by Tseng et al^7^ who analyzed the Surveillance, Epidemiology, and End Results database and showed that there was no difference in disease-specific survival when “less” radical was compared with “more” radical surgery. Nevertheless, no information on DFS and few patient characteristics (such as LVSI, margins status, and depth of invasion) were reported. In contrast, Derks et al^8^ published a retrospective study analyzing survival outcomes of patients with early cervical cancer treated with “less” vs “more” radical surgery in 3 referral hospitals in the Netherlands. After a propensity score matching analysis, the authors concluded that more RH was associated with better DFS in patients with tumor diameters between 2 and 4 cm and >4 cm but not in patients with tumors <2 cm. However, here, the surgery was retrospectively classified according to the Leiden TNM-like classification, the 2 study populations had some differences in baseline prognostic factors (although the incidence of metastatic lymph nodes was similar), and patients were included over a 30-year period.

The rationale behind these studies was that more radical surgery may be able to remove occult extracervical disease. However, in patients with small tumor diameter (≤20 mm), it is unlikely that the extent of parametrectomy had any effect on survival, as reported by the results from Derks et al.^8^ The risk of occult parauterine metastasis has been reported to be 2.1% to 31% and strictly related to tumor diameter.^22-28^ The combination of low incidence of occult parauterine metastasis and good prognosis in patients with small tumors may explain the lack of survival difference when comparing the different extents of RH in small-volume tumors.

A randomized trial comparing “simple” vs “radical” hysterectomy (NCT01658930—Radical Versus Simple Hysterectomy and Pelvic Node Dissection With Low-risk Early Stage Cervical Cancer [SHAPE] trial) in patients with low-risk early-stage cervical cancer has concluded enrollment and is awaiting completion of follow-up.^29^ More recently, the CONCERV trial was published: this study aimed to evaluate the feasibility of conservative surgery (conization or “simple” hysterectomy) in women with early-stage, low-risk cervical cancer.^30^ With a 3.5% cumulative incidence of recurrence, the authors concluded that select patients with early-stage, low-risk cervical carcinoma may be treated with nonradical surgery. The results from the CONCERV trial can be compared with the results of this study in which no difference in low-risk tumors (in our study reported as <2 cm tumors) could be found if more RH was performed.

### Clinical implications—the meaning of the study

The extent of RH was associated with prolonged DFS in early-stage cervical cancer. For this reason, referral to large-volume tertiary centers remains an important issue when dealing with patients with cervical cancer.^31^ Moreover, this highlights the importance of adequate training on RH^32^ to future generations of gynecologic oncology surgeons who will need to continue performing tailored radical surgery based on tumor characteristics.

Another clinical implication from the current study is the importance of tailoring the radicality of hysterectomy according to tumor characteristics as recommended by the European Society of Gynaecological Oncology guidelines^2^ to avoid a higher extent of radicality in small or low-risk tumors and vice versa. Preoperative assessment of tumor characteristics is crucial to deliver an adequate treatment.

### Research implications—unanswered questions

Further research should focus on the role of more extended radicality in patients with large and high-risk tumors, particularly in cases where no adjuvant treatment is administered. Furthermore, studies focusing on understanding of RH nomenclature are encouraged.

### Strengths and limitations

The main strength of this study is that it includes patients from 20 international referral centers, collecting data on the radicality of the surgery categorized according to a standardized classification with a relatively low incidence of adjuvant treatment. In addition, the perioperative management of the included patients followed national and international guidelines. Lastly, we included patients operated in a relatively recent and short period (10 years from 2007 to 2016).

We must acknowledge the few limitations. First, this study is retrospective. Furthermore, no information on the depth of stromal infiltration was reported. The number of patients with pretreatment suspicious parametrial involvement was not documented. Moreover, we lack perioperative morbidity outcomes. Imaging for assessment of metastatic disease was not standardized. There was no information on the frequency of surveillance or practice patterns to detect recurrences. Lastly, there is a potential classification bias, as defining the various types of RH is a challenge for any surgeon and what is considered type C by one might be considered type B by another and vice versa.

### Conclusions

In a population of patients with early-stage cervical cancer undergoing radical surgery, no survival difference was associated with more RH in tumors ≤20 mm. In contrast, a DFS improvement was observed in patients with tumors between 21 and 40 mm undergoing non–nerve-sparing RH. Therefore, type C2 RH should be preferred in this subgroup of patients. No difference in the pattern of recurrence according to the extent of radicality was observed. Non–nerve-sparing RH was associated with better DFS than nerve-sparing RH in patients with similar tumor characteristics after propensity match analysis.

## Supplementary Material

1

2

3

## Figures and Tables

**TABLE 1:** Distribution of demographical and clinical variables according to type of RH

Characteristic	Total (N=1257)	Nerve sparing RH (n=883)	Non-nerve sparing RH (n=374)	*P* value
Age (y)				.090
≤45	616 (49.0)	419 (47.5)	197 (52.7)	
>45	641 (51.0)	464 (52.5)	177 (47.3)	
Pathologic stage				<.0001
IB1	1186 (94.4)	846 (95.8)	340 (90.9)	
IIA1	71 (5.6)	37 (4.2)	34 (9.1)	
Grade				.259
1	120 (9.5)	92 (10.4)	28 (7.5)	
2	877 (69.8)	612 (69.3)	265 (70.9)	
3	260 (20.7)	179 (20.3)	81 (21.7)	
LVSI				<.0001
No	600 (47.7)	403 (45.6)	197 (52.7)	
Yes	432 (34.4)	291 (33.0)	141 (37.7)	
Unknown	225 (17.9)	189 (21.4)	36 (9.6)	
Histology				.291^a^
Squamous	823 (65.5)	566 (64.1)	257 (68.7)	
Adenocarcinoma	348 (27.7)	253 (28.7)	95 (25.4)	
Adenosquamous	67 (5.3)	50 (5.7)	17 (4.5)	
Others	19 (1.5)	14 (1.5)	5 (1.4)	
Diameter				<.0001
≤20 mm	712 (56.6)	535 (60.6)	177 (47.3)	
21–40 mm	545 (43.4)	348 (39.4)	197 (52.7)	
Adjuvant therapy				.633
No	909 (72.3)	642 (72.7)	267 (71.4)	
Yes	348 (27.7)	241 (27.3)	107 (28.6)	

*LVSI*, lymphovascular space invasion; *RH*, radical hysterectomy.

a The test was performed on squamous vs adenocarcinoma vs adenosquamous.

**TABLE 2:** Cox regression univariate and multivariate analysis for DFS on the entire group of 1257 patients

	Univariate	Multivariate (all variables)
DFS	HR (95% CI)	HR (95% CI)
Age (y)	*P*=.057	*P*=.43
≤45	1.00	1.00
>45	1.44 (0.99–2.11)	1.17 (0.79–1.74)
Stage	*P*=.003	*P*=.055
1b1	1.00	1.00
2a1	2.39 (1.34–4.25)	1.85 (0.99–3.39)
LVSI	*P*<.0001	*P*=.004
No	1.00	1.00
Yes	2.38 (1.58–3.57)	1.81 (1.16–2.82)
Unknown	0.90 (0.48–1.68)	0.74 (0.39–1.39)
Grade	*P*=.044	*P*=.34
1	1.00	1.00
2	1.72 (0.75–3.94)	1.68 (0.72–3.94)
3	2.60 (1.08–6.23)	1.96 (0.79–4.85)
Histology	*P*=.002	*P*=.002
SCC	1.00	1.00
Adenocarcinoma	1.02 (0.66–1.58)	1.43 (0.90–2.26)
Other	2.58 (1.51–4.40)	2.61 (1.51–4.48)
Adjuvant therapy	*P*<.0001	*P*=.95
No	1.00	1.00
Yes	2.01 (1.38–2.92)	0.97 (0.63–1.50)
Diameter	*P*<.0001	*P*<.0001
≤20 mm	1.00	1.00
21–40 mm	3.25 (2.17–4.87)	2.99 (1.96–4.59)
Radicality of RH	*P*=.049	*P*=.004
Nerve sparing	1.00	1.00
Non-nerve sparing	0.63 (0.40–0.99)	0.50 (0.31–0.81)

*CI*, confidence interval; *DSF*, disease-free survival; *HR*, hazard ratio; *LVSI*, lymphovascular space invasion; *RH*, radical hysterectomy; *SCC*, squamous cell carcinoma.

**TABLE 3:** Distribution of demographical and clinical variables according to type of RH (after propensity matching analysis)

Variable	Total (N=738)	Nerve-sparing RH (N=369)	Non–nerve-sparing RH (N=369)	*P* value
Age (y)				.82
≤45	391 (53.0)	197 (53.4)	194 (52.6)	
>45	347 (47.0)	172 (46.6)	175 (47.4)	
Pathologic stage				.67
IB1	683 (92.5)	343 (93.0)	340 (92.1)	
IIA1	55 (7.5)	26 (7.0)	29 (7.9)	
Grade				.21
1	70 (9.5)	42 (11.4)	28 (7.6)	
2	511 (69.2)	251 (68.0)	260 (70.5)	
3	157 (21.3)	76 (20.6)	81 (22.0)	
LVSI				.97
No	385 (52.2)	191 (51.8)	194 (52.6)	
Yes	281 (38.1)	142 (38.5)	139 (37.7)	
Unknown	72 (9.8)	36 (9.8)	36 (9.8)	
Histology				.91
Squamous	500 (67.8)	247 (66.9)	253 (68.6)	
Adenocarcinoma	190 (25.7)	96 (26.0)	94 (25.5)	
Adenosquamous	38 (5.1)	21 (5.7)	17 (4.6)	
Others	10 (1.4)	5 (1.4)	5 (1.4)	
Diameter				.82
≤20 mm	351 (47.6)	174 (47.2)	177 (48.0)	
21–40 mm	387 (52.4)	195 (52.8)	192 (52.0)	
Adjuvant therapy				.57
No	521 (70.6)	257 (69.6)	264 (71.5)	
Yes	217 (29.4)	112 (30.4)	105 (28.5)	

*LVSI*, lymphovascular space invasion; *RH*, radical hysterectomy.

## AJOG at a Glance

### Why was this study conducted?

International guidelines recommend tailoring the radicality of hysterectomy according to the known preoperative tumor characteristics in patients with early-stage cervical cancer. However, the survival benefit associated with the extent of radical hysterectomy (RH) is still a matter of debate.

### Key findings

Non–nerve–sparing RH was associated with improved 5-year disease-free survival (DFS) compared with nerve-sparing RH and represented an independent protective factor for risk of recurrence. Non–nerve-sparing RH was associated with better 5-year DFS in patients with tumors between 21 and 40 mm.

### What does this add to what is known?

In patients with early-stage cervical cancer, the extent of RH was associated with DFS improvement in patients with tumors between 21 and 40 mm but not in patients with tumors ≤20 mm.
