# Supplementary material for: Survival associated with extent of radical hysterectomy in early-stage cervical cancer: a subanalysis of the Surveillance in Cervical CANcer (SCCAN) collaborative study
Source: Am J Obstet Gynecol. Author manuscript; Available in PMC 2024 Mar 27. (PMC10966343; doi:10.1016/j.ajog.2023.06.030)
Supplement: 2 [file NIHMS1973095-supplement-2.pptx]

## Slide 1
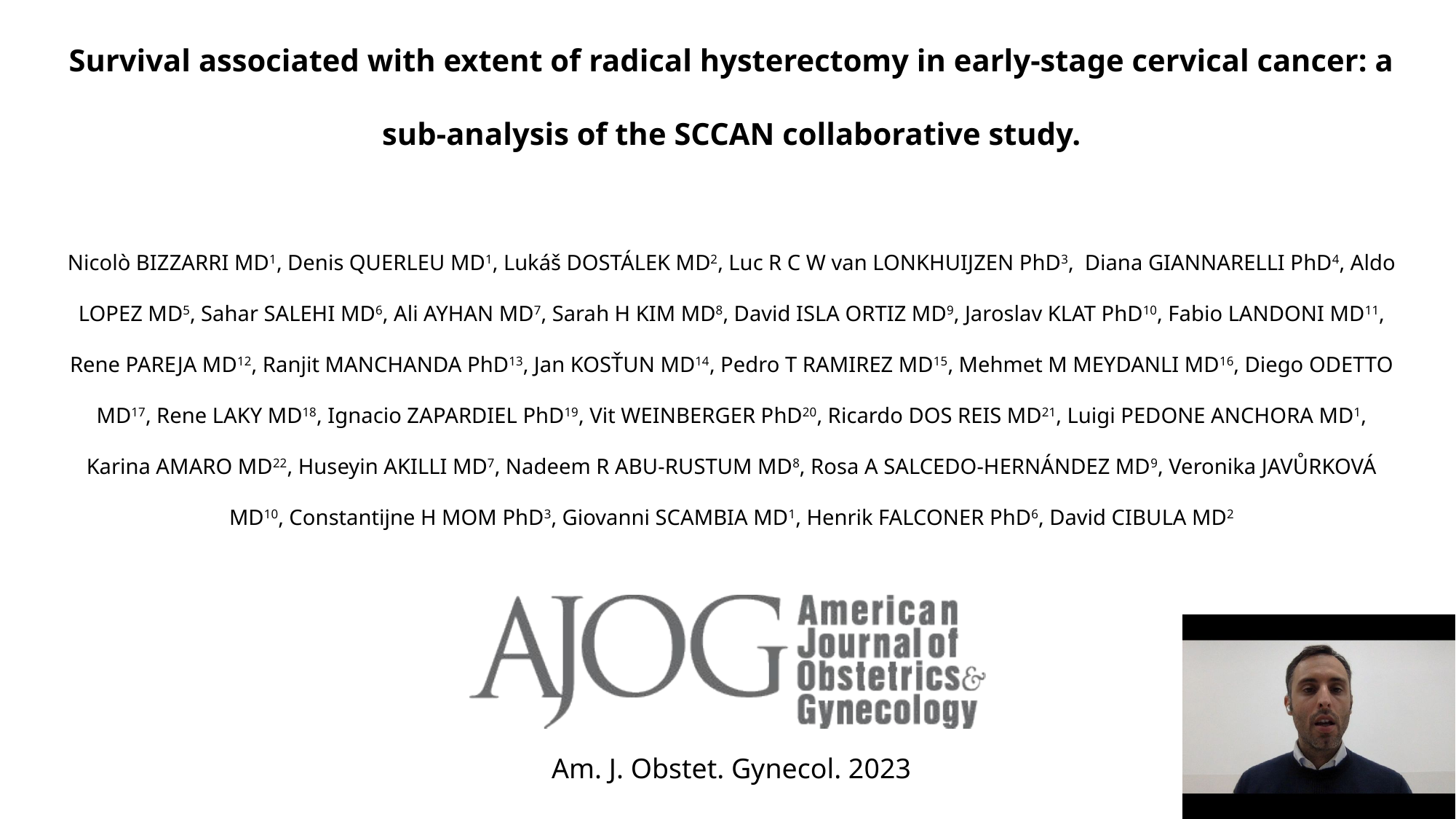

Survival associated with extent of radical hysterectomy in early-stage cervical cancer: a sub-analysis of the SCCAN collaborative study.
Nicolò BIZZARRI MD1, Denis QUERLEU MD1, Lukáš DOSTÁLEK MD2, Luc R C W van LONKHUIJZEN PhD3, Diana GIANNARELLI PhD4, Aldo LOPEZ MD5, Sahar SALEHI MD6, Ali AYHAN MD7, Sarah H KIM MD8, David ISLA ORTIZ MD9, Jaroslav KLAT PhD10, Fabio LANDONI MD11, Rene PAREJA MD12, Ranjit MANCHANDA PhD13, Jan KOSŤUN MD14, Pedro T RAMIREZ MD15, Mehmet M MEYDANLI MD16, Diego ODETTO MD17, Rene LAKY MD18, Ignacio ZAPARDIEL PhD19, Vit WEINBERGER PhD20, Ricardo DOS REIS MD21, Luigi PEDONE ANCHORA MD1, Karina AMARO MD22, Huseyin AKILLI MD7, Nadeem R ABU-RUSTUM MD8, Rosa A SALCEDO-HERNÁNDEZ MD9, Veronika JAVŮRKOVÁ MD10, Constantijne H MOM PhD3, Giovanni SCAMBIA MD1, Henrik FALCONER PhD6, David CIBULA MD2
Am. J. Obstet. Gynecol. 2023

## Slide 2
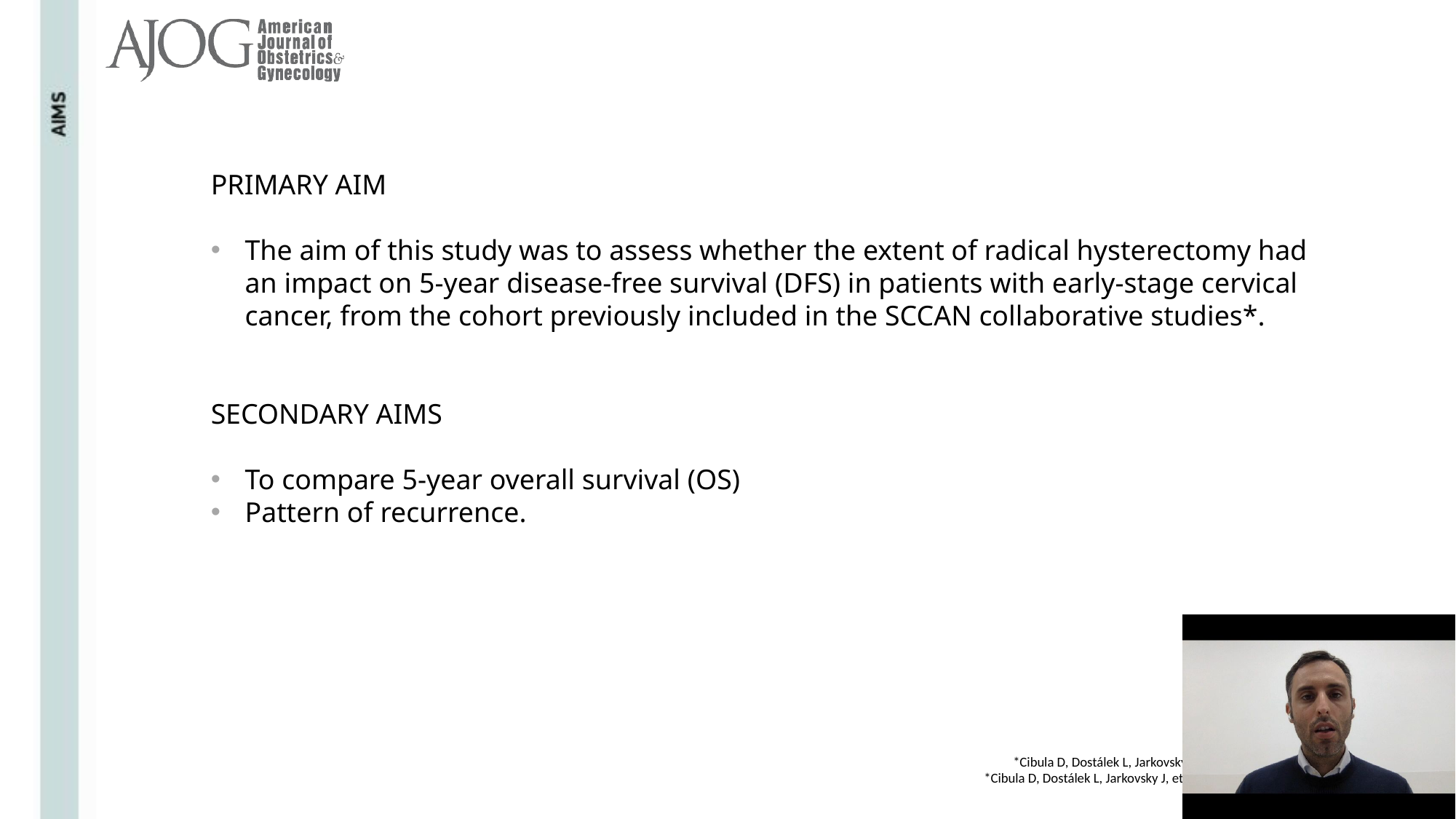

PRIMARY AIM
The aim of this study was to assess whether the extent of radical hysterectomy had an impact on 5-year disease-free survival (DFS) in patients with early-stage cervical cancer, from the cohort previously included in the SCCAN collaborative studies*.
SECONDARY AIMS
To compare 5-year overall survival (OS)
Pattern of recurrence.
*Cibula D, Dostálek L, Jarkovsky J, et al. Eur J Cancer. 2021;158:111-122
*Cibula D, Dostálek L, Jarkovsky J, et al. Gynecol Oncol. 2022;164(2):362-369

## Slide 3
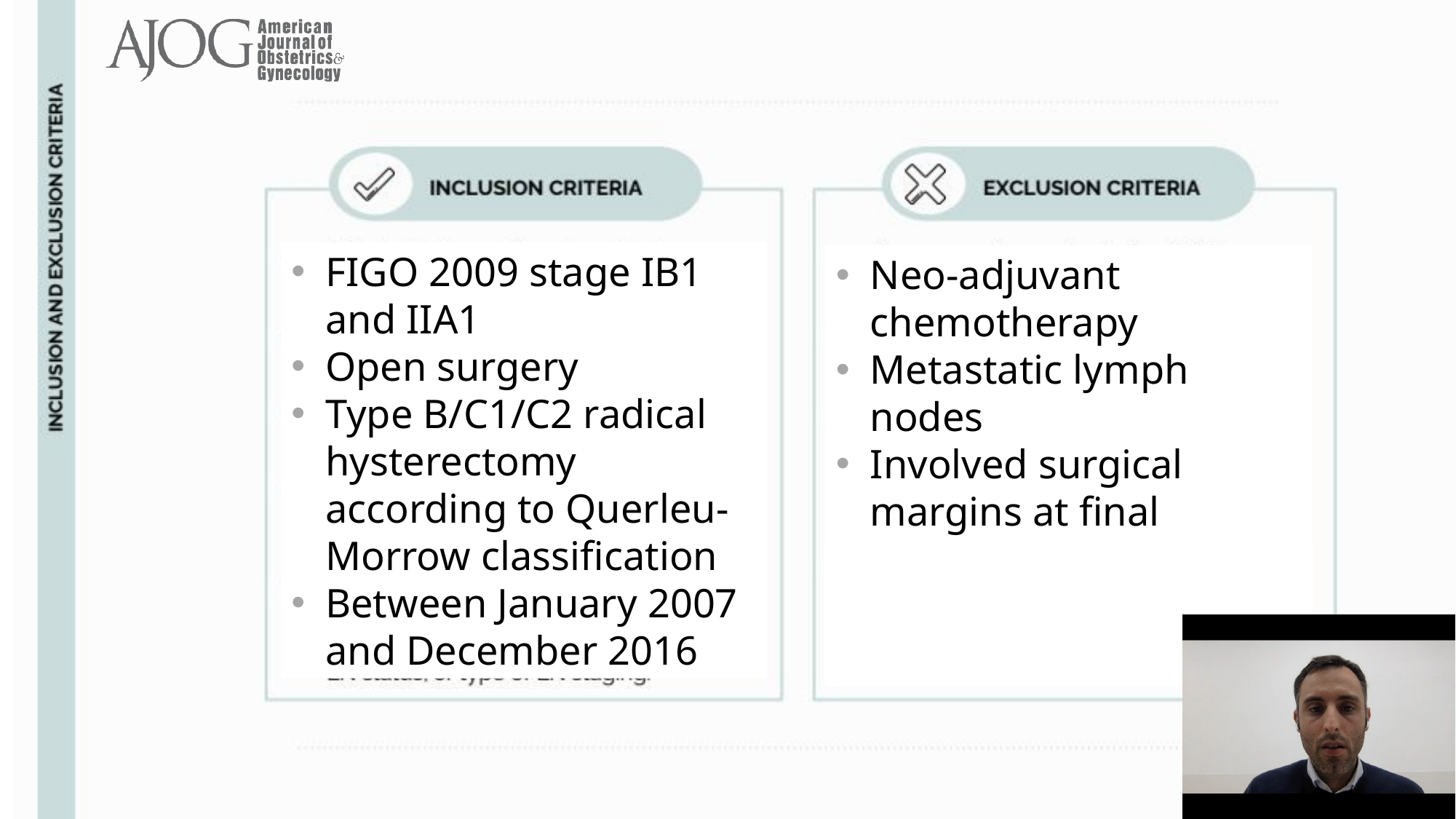

FIGO 2009 stage IB1 and IIA1
Open surgery
Type B/C1/C2 radical hysterectomy according to Querleu-Morrow classification
Between January 2007 and December 2016
Neo-adjuvant chemotherapy
Metastatic lymph nodes
Involved surgical margins at final histology

## Slide 4
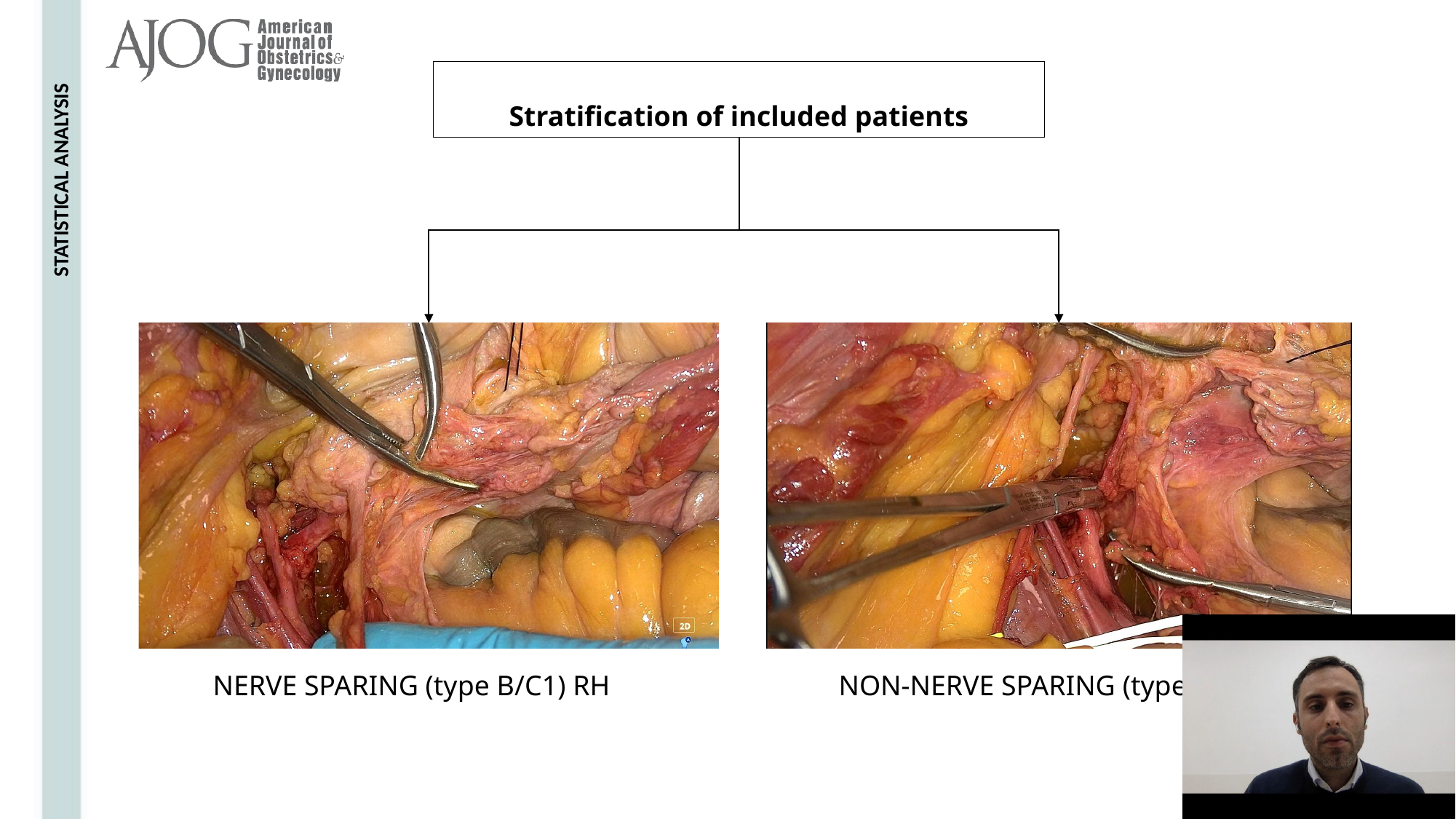

Stratification of included patients
STATISTICAL ANALYSIS
NERVE SPARING (type B/C1) RH
NON-NERVE SPARING (type C2) RH

## Slide 5
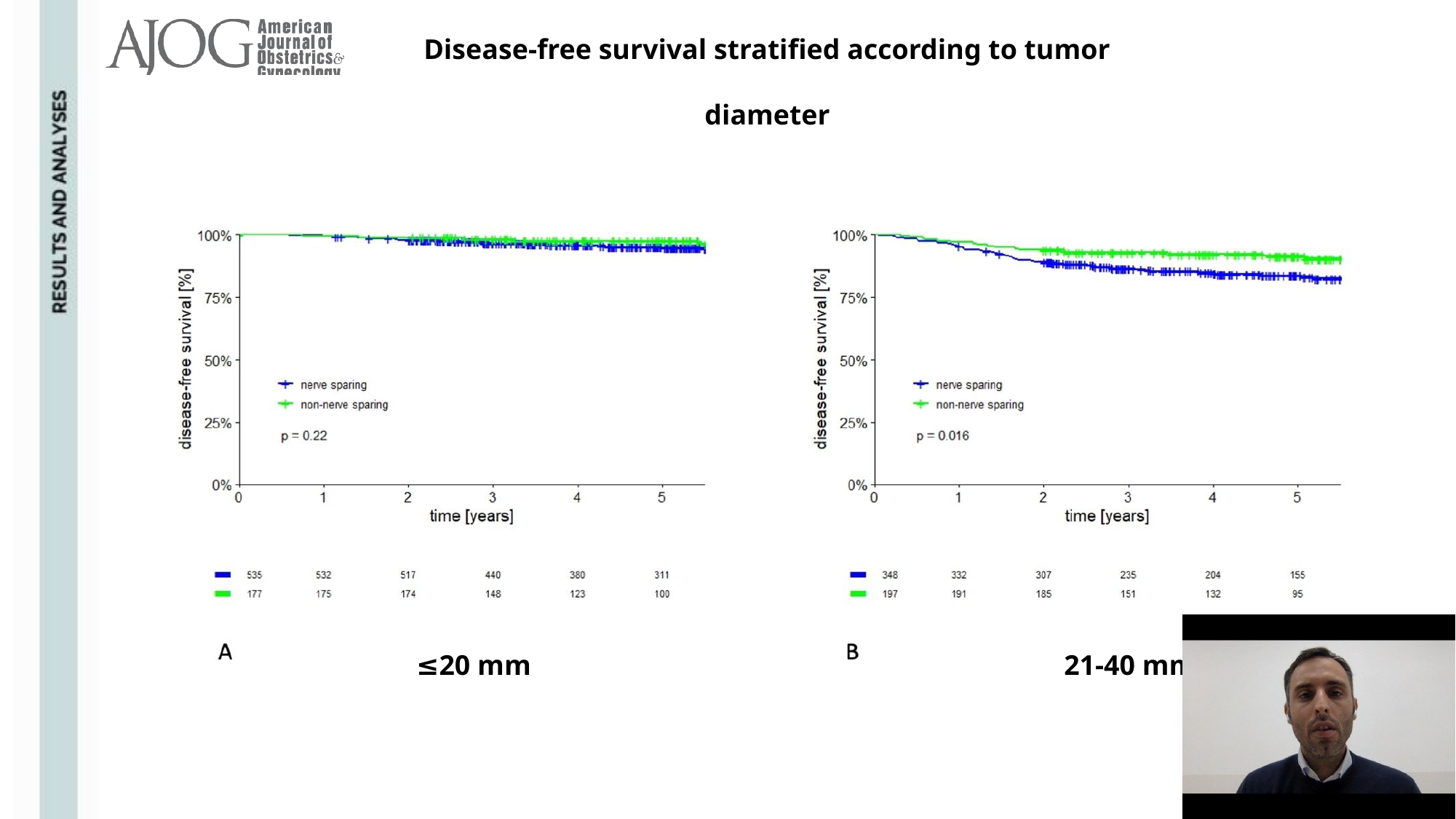

Disease-free survival stratified according to tumor diameter
21-40 mm
≤20 mm

## Slide 6
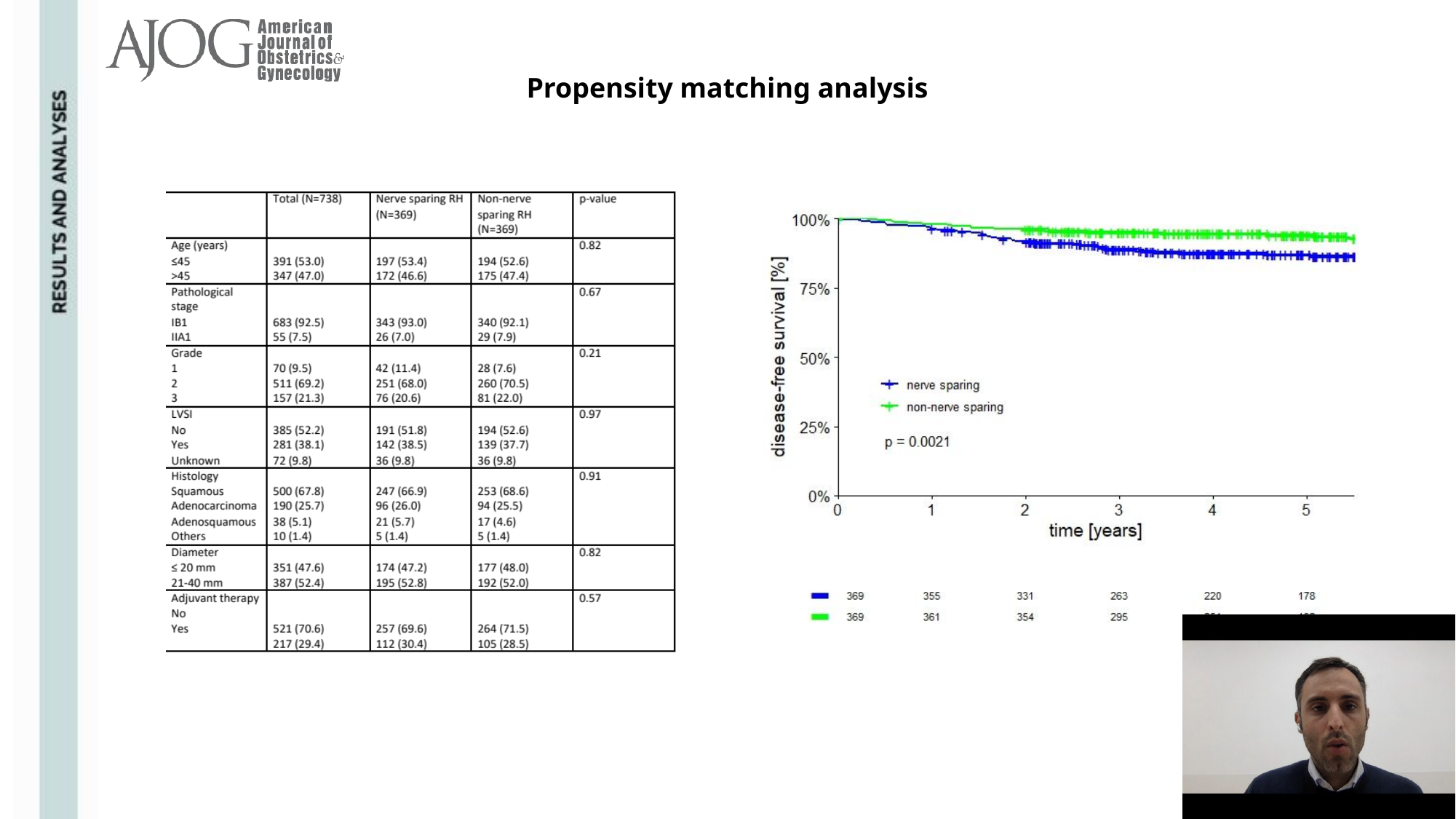

Propensity matching analysis

## Slide 7
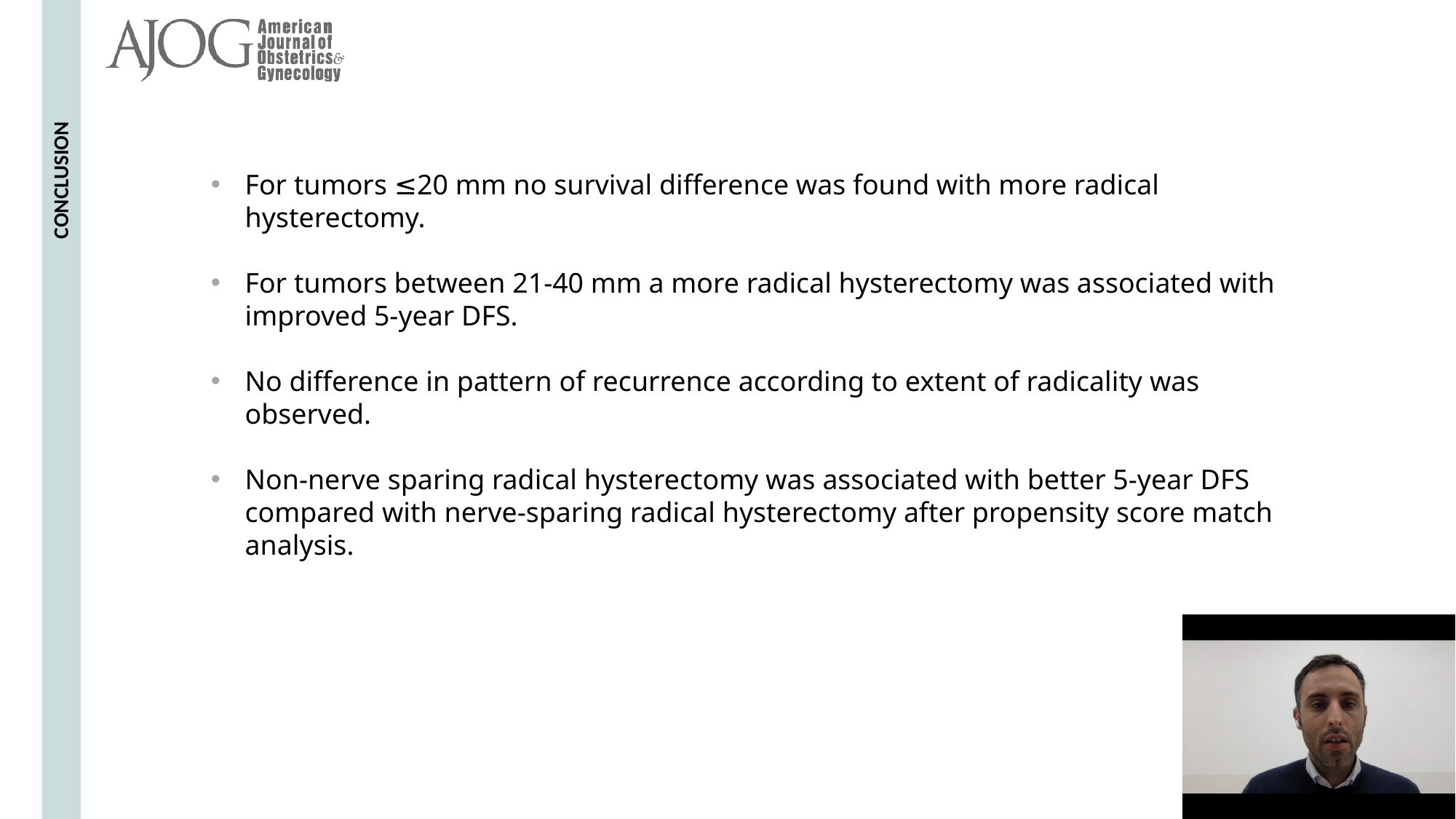

CONCLUSION
For tumors ≤20 mm no survival difference was found with more radical hysterectomy.
For tumors between 21-40 mm a more radical hysterectomy was associated with improved 5-year DFS.
No difference in pattern of recurrence according to extent of radicality was observed.
Non-nerve sparing radical hysterectomy was associated with better 5-year DFS compared with nerve-sparing radical hysterectomy after propensity score match analysis.

## Slide 8
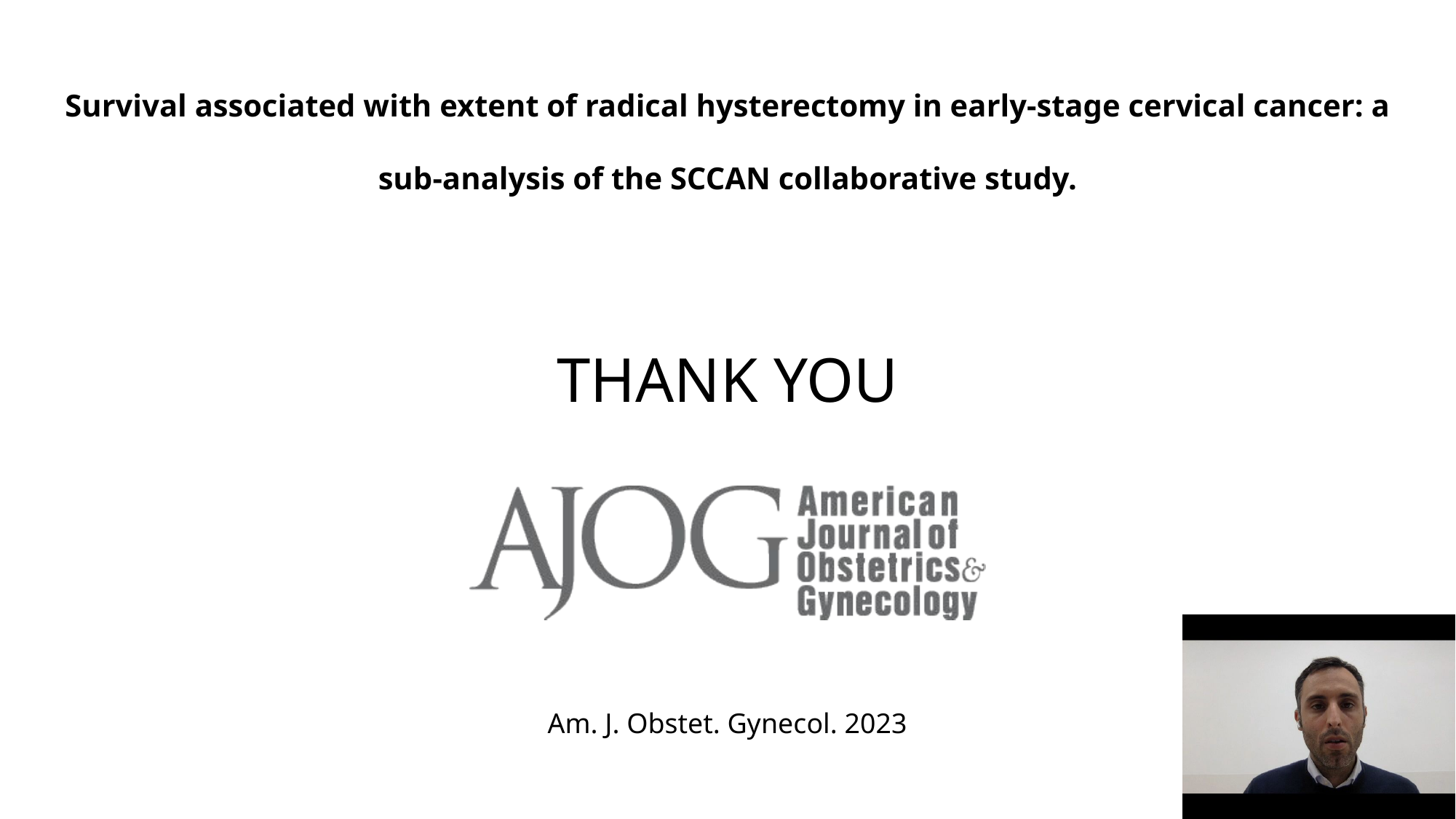

Survival associated with extent of radical hysterectomy in early-stage cervical cancer: a sub-analysis of the SCCAN collaborative study.
THANK YOU
Am. J. Obstet. Gynecol. 2023
