# Supplementary material for: Survival associated with extent of radical hysterectomy in early-stage cervical cancer: a subanalysis of the Surveillance in Cervical CANcer (SCCAN) collaborative study
Source: Am J Obstet Gynecol. Author manuscript; Available in PMC 2024 Mar 27. (PMC10966343; doi:10.1016/j.ajog.2023.06.030)
Supplement: 3 [file NIHMS1973095-supplement-3.pdf]

SUPPLEMENTAL TABLE 1

Cox regression univariate and multivariate analyses for overall survival on the entire group of 1257 patients

	Univariate	Multivariate (all variables)
OS	HR (95% CI)	HR (95% CI)
Age (y)	*P*=.096	*P*=.50
≤45	1.00	1.00
>45	1.59 (0.92—2.73)	1.21 (0.69—2.14)
Stage	*P*=.018	*P*=.30
1b1	1.00	1.00
2a1	2.61 (1.18—5.78)	1.57 (0.67—3.65)
LVSI	*P*=.002	*P*=.11
No	1.00	1.00
Yes	2.40 (1.35—4.27)	1.66 (0.88—3.11)
Unknown	0.77 (0.31—1.94)	0.71 (0.28—1.80)
Grade	*P*=.038	*P*=.22
1	1.00	1.00
2	2.45 (0.59—10.16)	2.15 (0.50—9.19)
3	4.50 (1.04—19.48)	3.16 (0.70—14.23)
Histology	*P*=.20	*P*=.35
SCC	1.00	1.00
Adenocarcinoma	0.83 (0.44—1.57)	1.17 (0.61—2.27)
Other	1.89 (0.84—4.26)	1.83 (0.80—4.18)
Adjuvant therapy	*P*<.0001	*P*=.17
No	1.00	1.00
Yes	2.64 (1.56—4.48)	1.53 (0.83—2.82)
Diameter	*P*<.0001	*P*=.09
≤20 mm	1.00	1.00
21—40 mm	2.14 (1.25—3.67)	1.63 (0.92—2.89)
Class	*P*=.78	*P*=.46
Nerve sparing	1.00	1.00
Nonnerve sparing	0.92 (0.50—1.69)	0.79 (0.42—1.47)

CI, confidence interval; *DSF*, disease-free survival; *HR*, hazard ratio; *LVSI*, lymphovascular space invasion; *OS*, overall survival; *RH*, radical hysterectomy.

Bizzarri. Survival after different extent of radical hysterectomy in early-stage cervical cancer. Am J Obstet Gynecol 2023.

SUPPLEMENTARY TABLE 2

Pattern of recurrence in patients undergoing nerve-sparing vs non—nerve-sparing radical hysterectomy

Variable	Nerve sparing (n=88)	Nonnerve sparing (n=23)	*P* value
Isolated	53 (60.2)	18 (78.3)	.14[a]
Multiple	33 (37.5)	5 (21.7)	
Unknown	2 (2.3)	0 (0)	
Combined	19 (21.6)	5 (21.7)	.99[a]
Distant	29 (33.0)	8 (34.8)	
Pelvic	38 (43.2)	10 (43.5)	
Unknown	2 (2.3)	0 (0)	
Sites			
Pelvic central	34	10	
Pelvic lateral	11	2	
Pelvic other	4	3	
Abdominal liver	7	4	
Abdominal other	18	5	
Thorax lungs	16	6	
Thorax other	3	1	
Bones	4	0	
Brain	2	0	
Scar port	1	0	
Ovary	1	0	
Other distant	5	1	

[a] Calculated without considering unknown values.

Bizzarri. Survival after different extent of radical hysterectomy in early-stage cervical cancer. Am J Obstet Gynecol 2023.

SUPPLEMENTARY TABLE 3

Pattern of recurrence in patients undergoing nerve-sparing vs non–nerve-sparing radical hysterectomy after propensity match analysis

Variables	Nerve sparing (n=48)	Nonnerve sparing (n=23)	*P* value
Isolated	34 (70.8)	18 (78.3)	.69[a]
Multiple	12 (25.0)	5 (21.7)	
Unknown	2 (4.2)	0 (0)	
Combined	8 (16.7)	5 (21.7)	.70[a]
Distant	13 (27.1)	8 (34.8)	
Pelvic	25 (52.1)	10 (43.5)	
Unknown	2 (4.2)	0 (0)	
Sites			
Pelvic central	18	10	
Pelvic lateral	6	2	
Pelvic other	0	3	
Abdominal liver	1	4	
Abdominal other	11	5	
Thorax lungs	6	6	
Thorax other	0	1	
Bones	1	0	
Brain	1	0	
Scar port	1	0	
Other distant	2	1	

[a] Calculated without considering unknown values.

Bizzarri. Survival after different extent of radical hysterectomy in early-stage cervical cancer. Am J Obstet Gynecol 2023.
